# Ameliorating effect of the biological Zinc nanoparticles in abamectin induced hepato-renal injury in a rat model: Implication of oxidative stress, biochemical markers and COX-2 signaling pathways

**DOI:** 10.3389/fphar.2022.947303

**Published:** 2022-09-12

**Authors:** Ahmed A. A. Aioub, Sameh A. Abdelnour, Mustafa Shukry, Ahmed M. Saad, Mohamed T. El-Saadony, Zhongli Chen, Ahmed E. A. Elsobki

**Affiliations:** ^1^ Plant Protection Department, Faculty of Agriculture, Zagazig University, Zagazig, Egypt; ^2^ Animal Production Department, Faculty of Agriculture, Zagazig University, Zagazig, Egypt; ^3^ Department of Physiology, Faculty of Veterinary Medicine, Kafrelsheikh University, Kafrelsheikh, Egypt; ^4^ Biochemistry Department, Faculty of Agriculture, Zagazig University, Zagazig, Egypt; ^5^ Department of Agricultural Microbiology, Faculty of Agriculture, Zagazig University, Zagazig, Egypt; ^6^ Key Laboratory of the Three Gorges Reservoir Eco-Environment, Chongqing University, Chongqing, China

**Keywords:** abamectin, Zinc nanoparticles, antioxidant, oxidative stress, immunohistochemical study

## Abstract

Extensive use of abamectin (ABM) as an anthelmintic in veterinary systems adversely affects the health and welfare of animals and humans. Zinc nanoparticles (ZnNPs) have therapeutic benefits and ameliorate the effect of environmental pollutants. In this study, we assessed the ameliorative effect of ZnNPs against the sub-lethal toxicity of ABM in rats. Forty healthy rats were randomly selected into four groups (*n* = 10); the control received normal saline and test rats were treated orally twice weekly with ABM (1 mg/kg bwt), ZnNPs (10 mg/kg bwt) and ABM + ZnNPs for 28 days. Upon completion of the study period, blood and tissue samples were collected and prepared for hematological, biochemical, pathological, and immunohistochemical analysis. Our results showed that ABM treatment significantly decreased body weight gain (BWG), red blood cells (RBCs), hemoglobin (Hb), hematocrit (HC), and platelet (PLT); while it significantly increased white blood cells (WBCs) and lymphocytes. ABM also significantly decreased antioxidant enzyme activities: superoxide dismuthase (SOD), glutathione peroxidase (GPx), and catalase (CAT) and increased hydrogen peroxide and malondialdehyde levels compared with other groups. ABM significantly raised alanine aminotransferase (ALT), aspartate amino transaminase (AST), and alkaline phosphatase (ALP) levels, which was restored by co-administration of ZnNPs. Moreover, ZnNPs ameliorated ABM-mediated negative histopathological changes in the liver and kidney tissues, exhibiting a significant protective effect. Cyclooxygenase 2 (COX-2) + immuno-expression were reduced after pretreatment with ZnNPs. These findings suggested that co-administration of ZnNPs with ABM mitigated its toxicity by combating oxidative stress and boosting antioxidant capacity, indicating the efficacy of ZnNPs in attenuating ABM toxicity.

## Graphical Abstract

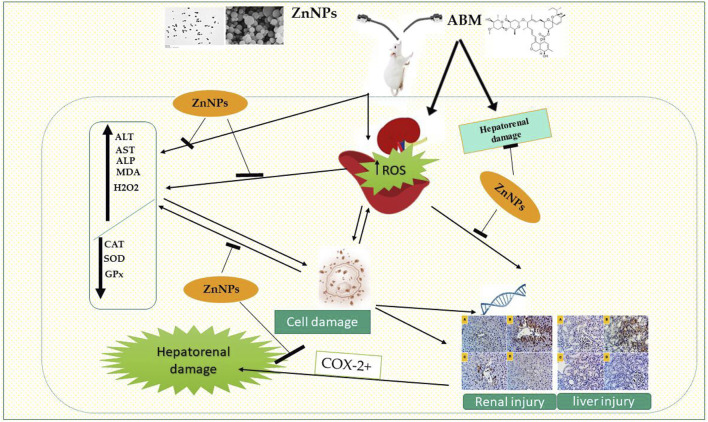

## Introduction

Pesticides are extensively used in veterinary medicine and agriculture systems for controlling pests and for boosting crop productivity, respectively (Sharma et al., 2017). However, widespread usage of these agrochemicals has become a global issue as its non-targeted mechanism makes it unsafe for humans and the environment (Hong et al., 2020). Absorption of these chemicals by living organisms might cause kidney and liver dysfunction, presenting a significant health concern to animals. Additionally, these pesticides and their metabolites mostly end up in rivers and estuaries where they might be hazardous to wildlife (El-Shenawy, 2010). Abamectin (ABM) is a pesticide that is commonly used in veterinary medicine because of its efficacy and pharmacological importance (Mahmoud et al., 2021). ABM is a fermented product generated by the soil actinomycete (*Streptomyces avemitilis*) (Burg and Stapley, 1989). The avermectins groups in ABM (80% avermectin B1a and 20% B1b) exhibit high similarity in bioactivity and toxicological relevance (Casali-Pereira et al., 2015).

Additionally, ABM has been promoted as an acaricide and insecticide for crops, fruits, and vegetables, and has been extensively used as an anthelmintic to treat animal diseases (Bebe and Panemangalore, 2003; Kolar et al., 2008). ABM is a neurotoxin that acts via the glutamate and γ-amino butyric acid-gated chloride channels found in neurons that are protected by the blood-brain barrier in living organisms (Omura, 2008; Ishaaya, 2012). Although, at appropriate dosage, ABM has been found to be safe for animals and humans, its presence in different ecosystems is potentially hazardous for various animal and agricultural ecosystems. Several studies have shown that ABM degrades slowly because of its water insolubility and lipophilicity (Herd, 1995; Kolar et al., 2008). Previous studies also demonstrated that ABM impairs antioxidant function by boosting reactive oxygen species (ROS) synthesis (Zhang et al., 2017; Radi et al., 2020). Moreover, it reduces immune capacity, thus triggering immunodepression in animals (Wang et al., 2011). Rats exposed to ABM exhibit significant ROS production in both hepatic and cerebral tissues (Radi et al., 2020). Furthermore, it can trigger hepatotoxic, renal toxic, neurotoxic, and genotoxic effects in both target and non-target organisms (Yoon et al., 2004; Zhang et al., 2017; Mossa et al., 2018).

Rats exposed to high levels of avermectins exhibit significantly elevated serum aspartate aminotransferase (AST), alanine aminotransferase (ALT), alkaline phosphatase (ALP) levels accompanied by reduction in the levels of antioxidant enzymes such as superoxide dismutases (SOD), catalase (CAT), and glutathione peroxidase (GPx) in liver and kidney tissues (Bebe and Panemangalore, 2003). Previous studies showed that insecticides might induce alterations in enzyme activity linked to antioxidant defense machineries (Gultekin et al., 2001; Ranjbar et al., 2002). Zinc (Zn) is a trace mineral essential for cell development and differentiation. It is a vital component for combating free radical synthesis, modulating immune functions, and protecting from injury (Sheikh et al., 2010). Zn exhibits several pharmacological properties such as antioxidant, anti-viral, anti-inflammatory, and anti-fungal activities (Prasad, 2014). Additionally, Zn is critical for maintaining the structure and function of biomembranes. It also aids the activity of several antioxidant enzymes involved in cellular defense (Mansour et al., 2017; Lee, 2018).

Recently, there have been massive developments in nanotechnology with various potential applications. Nanoparticles are versatile tools interrelated with biological structures because of their large surface area and nano-size. Furthermore, they have been proven to dramatically improve the bioavailability and absorption of several medications (Yuan et al., 2008; Refat et al., 2021; Isaac et al., 2017; Najafi et al., 2020). Several studies have used Zn or selenium to ameliorate oxidative damage and hormonal imbalance mediated by ABM (Mansour et al., 2017) or cadmium (Liu et al., 2020) in rats. These elements were utilized to mitigate pesticide-induced toxicity and oxidative stress in living organisms. Due to the essential role of Zn in humans and its valuable biological properties, there is great interest in producing Zn nanoparticles (ZnNPs) using novel methods. Abdelnour et al. (2021) summarized the potential use of ZnNPs in livestock nutrition; showing improved performance, immunity, antioxidant capacity, and nutrient bioavailability in the animals. These nanoparticles also enhanced the quantity and quality of animal products by mitigating environment-associated risks. However, there is a lack of information regarding the use of ZnNPs as ameliorative agents against oxidative stress induced by ABM in hepato-renal tissues of rats. Therefore, we hypothesized that ZnNPs might neutralize the deleterious effects of ABM-induced toxicity in male rats by improving its antioxidant capacity and mitigating proinflammatory mediators.

## Materials and methods

Abamectin (Vertemic, 1.8% EC) was obtained from Syngenta Company for Agricultural Service, Egypt. Alanine amino transaminase (ALT), aspartate amino transaminase (AST), alkaline phosphatase (ALP), superoxide dismutase (SOD), and catalase (CAT), malondialdehyde (MDA), hydrogen peroxide (H_2_O_2_), and glutathione peroxidase (GPx) kits were purchased from Biodiagnostic Company (Dokki, Giza, Egypt).

### Isolation and identification of zinc resistant bacteria

The diary samples were obtained from a special farm. Milk samples were aseptically collected in sterile containers using clean gloves. The collected samples were stored at 4°C until transport to the laboratories of the Agricultural Microbiology Department, Faculty of Agriculture, Zagazig University, Egypt, for immediate processing. The diary samples were used to isolate lactic acid bacteria as follows: The collected diary samples were homogenized. In a scraw bottle, 10 mL of the homogenized sample was mixed in 90 mL of saline peptone buffer (0.1% peptone water + 0.85% salt) and stirred for 10 min at 25°C to prepare a 10^−1^ dilution. One ml of the previous dilution was added to a 9 mL buffer peptone tube to obtain a 10^−2^ dilution. Further serial dilution to 10^−7^. 100 μL of each dilution was spread across the surface of selective agar media in sterilized plastic petri dishes (90 mm diameter) using sterilized L-shaped spreaders. For each dilution, three plates were used for each sample. For the isolation of zinc resistant *Lactobacillus species* isolates, de Man Rogosa and Sharpe (MRS) agar medium supplemented with zinc nitrate (1, 3, and 5 mM) (CM0361, Oxoid Ltd., Basingstoke, Hampshire, United Kingdom) at pH 6.5 was used. The plates were incubated using AnaeroGen sacks (Oxoid Ltd.) at 37 ± 2°C in an anaerobic GasPak system (Becton Dickinson, NJ, United States) for 48 h. Colonies were randomly collected from each sample and purified using the streak plate technique on MRS agar medium. All bacterial cultures were kept at a temperature of 4°C until use. (El-Saadony et al., 2021a; Alagawany et al., 2021; El-Hack et al., 2021). Zinc resistant isolates were isolated, purified, and preserved at 4°C. According to Forouhandeh et al. (2010) , the efficiency of the isolate was specified using morphological, biochemical, and molecular assays described in Bergey’s Guide. MALDI TOF mass spectrometry was used to identify the strains (Karaduman et al., 2017).

#### Biosynthesis and characterization of biological zinc nitrate nanoparticles

The selected isolate was inoculated into 100 ml Luria-Bertani broth and incubated at 37°C with shaking until log phase. The mixture was centrifuged at 5,000 *x* g for 10 min, and then the bacterial supernatant was obtained. Then, 20 ml of this supernatant was mixed with 100 ml of enrichment medium (0.5 g sodium nitrate, 5 g sodium chloride, 0.1 g ammonium chloride, 2.7 g di-potassium hydrogen phosphate, 3 g tryptone, 1 g beef extract, 0.5 g yeast extract, and 3 g glucose in 1000 ml distilled water) supplemented with 5 mM of zinc nitrate, then incubated for 2 days at 35°C under shaking (150 rpm). The resultant white precipitate was indicated the biosyntheses of ZnNPs. The reaction mixture was centrifuged at 15,000 rpm for 15 min to obtain the precipitate. The zinc nanoparticles were collected, washed several times with distilled water, and lyophilized (Yusof et al., 2020).

The biological ZnNPs were characterized by UV–Vis spectroscopy using Laxco™, Alpha-1502 dual beam spectrophotometer (El-Saadony et al., 2020; El-Saadony et al., 2021b; Saad et al., 2021). TEM was used to evaluate the morphological properties of these ZnNPs (El-Saadony et al., 2021c; El-Hack et al., 2021). The functional groups in the ZnNPs suspension were estimated by Fourier transform infrared spectroscopy [FT-IR; JASCO (FTIR-6200)]. Dynamic light scattering (DLS) spectroscopy using Malvern Zetasizer Nano ZS was done to analyze the size distribution of the ZnNPs and their zeta potential (El-Saadony et al., 2019; El-Saadony et al., 2021d; El-Saadony et al., 2021e).

### Animals and experimental design

A total of 40 mature male Sprague Dawley rats (average initial weight: 160 ± 20 g; 13–15 weeks old) were obtained from the Laboratory Animal Housing Unit, Faculty of Veterinary Medicine, Zagazig University, Egypt. Animals were kept in a stainless-steel cage in a well-ventilated room with free access to food and water with 12 h light/12 h dark cycle. The experimental animals were acclimated to laboratory conditions for 1 week before starting the experiments.

The rats were randomly distributed into four experimental groups (*n* = 10); Group I: control group administered with normal saline; Group II: given ABM orally at a dosage of 1/10 LD_50_ (1 mg/kg b.wt) according to Meligi and Hassan (2017); Groups III: given ZnNPs (10 mg/kg b. wt.) 30 min after ABM administration; Group IV: given ZnNPs (10 mg/kg b. wt.) only. The ZnNPs dose was selected based on previous reports (Torabi et al., 2013; Bashandy et al., 2018). All groups were treated orally twice a week for 28 days. Rats were inspected daily and any clinical signs of toxicity were noted. The following equation was used to determine the body weight gain: body weight gain = [(final body weight−initial body weight)/initial body weight] ×100.

### Sampling

After 28 days of treatment, the animals were fasted overnight and then weighed. The blood samples were drawn from the rats in the control and treatment groups by putting them under ether anesthesia and puncturing the retro-orbital sinus with a fine antiseptic glass capillary. The samples were collected in tubes using 10% EDTA as an anticoagulant for hematological examination. An aliquot of the blood samples were kept in tubes and allowed to coagulate at room temperature for 30 min. Next, the samples were centrifuged for 20 min at 3,000 rpm. The resulting serum was stored at −20°C for further examination (antioxidant enzymes, oxidative stress measurements and liver enzymes). The livers and kidneys were extracted, saline-washed, and kept in neutral buffered formalin (10%) for histopathological and immunohistopathological examinations.

### Hematological parameters

The collected blood samples were used to determine the hematological parameters including leukogram and erythrogram profiles. The erythrogram profile including red blood cell count (RBC, 10^12^/L), hemoglobin level (Hb, g/L), hematocrit (HCT, %), mean corpuscular volume (MCV, fL), mean corpuscular hemoglobin (MCH, pg), mean corpuscular hemoglobin concentrations (MCHC, g/dL), platelet count (PLT, 10^9^/L), mean cell volume (MCV, fL), and the leukogram profile such as white blood cell count (WBC, 10^9^/L), lymphocyte percentage, and complete blood counts were evaluated using an automated blood cell analyzer (Hemascreen18, Hospitex diagnostics, Sesto Fiorentino, Italy).

### Antioxidant profile

SOD activity was evaluated using a method by Nishikimi et al. (1972) based on the capacity of SOD to restrict phenazine methosulphate intermediated nitro-blue tetrazolium dye reduction. CAT activity was measured in units per Gram of tissue (U/g) at 510 nm using Aebi (1984). GPx activity was assessed spectrophotometrically based on a previous study (Paglia and Valentine, 1967). GPx activity was calculated by oxidizing NADPH and GSH using glutathione reductase and then measuring the drop in absorbance at 340 nm, which was expressed in units/mg protein.

### Oxidative stress measurements

MDA were measured at 532 nm using the thiobarbituric acid assay established by Ohkawa et al. (1979) and was expressed as nmol/mg protein. H_2_O_2_ was measured based on the method by Pick (1980) at 610 nm.

### Liver function

Liver enzymes including AST and ALT were measured calorimetrically as reported previously by Reitman (1957) while the levels of alkaline phosphatase ALP was assessed using a method by Ellis et al. (1971).

### Histopathological examination

The liver and kidney samples were preserved in 10% formalin solution, dried, cleaned with xylene, and entrenched in paraffin using an automated tissue processor. Next, a rotary microtome was used to produce 5-μm thick slices, which were then stained with hematoxylin and eosin (Suvarna et al., 2018). Then, five stained liver and kidney sections per test rat were microscopically evaluated at various magnifications to assess qualitative histological fluctuations and for histomorphometric analysis. The histopathological abnormalities in the hepatic and renal tissues were graded by describing histomorphological changes in five fields per section for each studied organ.

### Immunohistochemical study

For immunohistochemical (IHC) analysis, COX-2 antigens were stained in the hepatic and renal tissues using rabbit monoclonal anti-COX-2 antibody (ab15191) (Abcam, United Kingdom), and 3,30-diaminobenzidine chromogen (DAB) according to the avidin-biotin-peroxidase complex procedure described by Su-Mingxhsu et al. (1981). Additionally, the negative controls were treated using phosphate buffer saline instead of primary antibodies to verify whether the IHC analysis is selective and can eliminate non-specific responses and false-positive results (Hewitt et al., 2014). The DAB density is not proportionate to the epitope concentration and most hepatic or renal cells were immune-positive at varying degrees for both biomarkers. Therefore, quantitative assessment of the COX-2 immunodepression was achieved by calculating the fractions of DAB brown spots to the overall image areas. Five fixed-size microscopic images/organs/animals were captured at the same magnification (×40) and exposure duration using open-source ImageJ program version 1.41.

### Statistical analysis

Statistical data were expressed as mean ± standard error of the mean unless otherwise stated. GraphPad Prism program v.8 (GraphPad Software Inc., La Jolla, CA, United States) was used for data analysis. The differences between groups were analyzed using one-way ANOVA using Tukey’s post-hoc test and statistically significant difference was considered at *p* < 0.05.

## Results

### Isolation and Identification of *Lactobacillus* isolate

Thirty-two isolates were obtained from diary sample mixtures on MRS plates and labeled as (TA1, TA2 … TA32). Sixteen isolates appeared in 1 mM Zinc nitrate-supplemented MRS, and nine isolates in 2 mM Zinc nitrate-supplemented MRS, while one isolate appeared at 5 mM Zinc nitrate-supplemented MRS. The zinc resistant isolate was served for the morphological, biochemical, and physiological identification in the Bergey Handbook, it was a Gram-positive, catalase-negative, and non-spore-forming bacterium. It did not produce NH_3_ from Arginine or gas from glucose, suggesting that this isolate is homo-fermentative. Isolate TA20 successfully grew at 15°C, but not at 45°C, and was tolerant to NaCl concentrations of 4 and 6.5%. The selected bacterium was similar to *Lactobacillus* spp. and categorized as *Lactobacillus fermentum* TA20, which was confirmed by MALDI TOF mass spectrometry analysis. These results showed that our isolate was 99% similar to *Lactobacillus* spp. and based on the MALDI TOF score, *Lactobacillus fermentum* TA20 is analogous to *Lactobacillus fermentum* 20063 DSM.

### Characterization of ZnNPs

**FIGURE 1:**
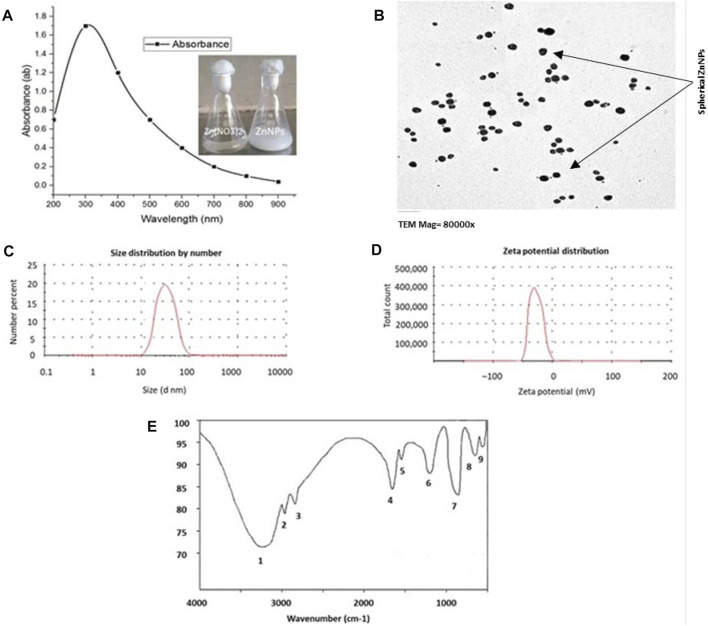
Characterization of ZnNPs synthesized by *Lactobacillus fermentum* TA20. **(A)** U.V. absorbance at 310 nm, **(B)** TEM image observes the average size of spherical ZnNPs, **(C)** Exact size of 51 nm by Zeta sizer, **(D)** Surface net negative charge by Zeta potential (-25.36 mV), and **(E)** Functional groups attached to ZnNPs detected by FTIR.

When the bacterial supernatant was added to the zinc nitrate solution and incubated at optimum conditions, the mixture turned white, indicating ZnNPs formation (Figure 1A). The biological ZnNPs absorbed ultraviolet light and showed sharp plasmon peaks at 310 nm with time dependent increase in absorbance (Figure 1A). The size and morphology of the ZnNPs were detected by TEM, which showed that ZnNPs were spherical in form with an average diameter of 45–75 nm (Figure 1B). The FTIR spectrum showed nine distinct peaks between 3,243.23 and 540.54 cm^−1^, indicating OH, NH, C=C, CO, CH, C-Cl groups. DLS analysis was done to measure the size and charge of ZnNPs, which were 51 nm (Figure 1C) and −25.36 mV (measured using zeta potential), respectively (Figure 1D). The attachment of the active groups, which are obtained from the bacterial supernatant and are responsible for stabilizing the NPs, to the ZnNPs surface was characterized by FTIR spectroscopy (Figure 1E).

### Effects on body weight gain and hematological parameters

**FIGURE 2:**
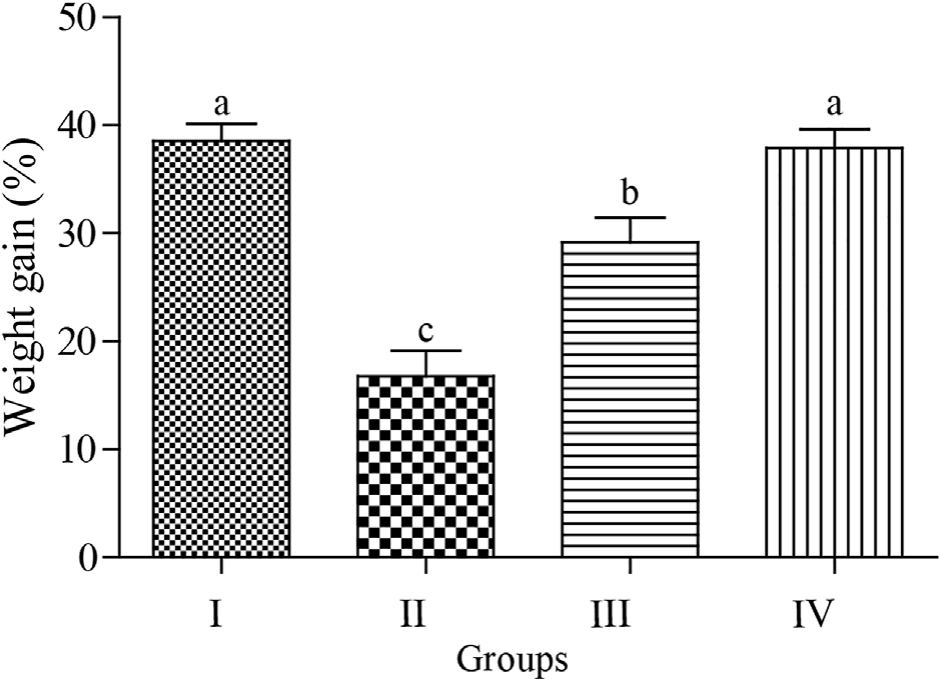
Effect of Abmectin (ABM; G II) and ZnNPs (ZnNPs; G IV) alone and together (G III) on for 3 weeks on rat’s body weight gain (%) compared with control (G I). The different letters represent the statistically significant differences at (*p* < 0.05) with mean +SD between control and treatment followed by Tukey’s post hoc test.

As shown in Figure 2, the body weight gain (BWG) was significantly reduced by exposure to acute ABM (*p* < 0.05) with the lowest and highest BWG values observed in the ABM and ZnNPs groups, respectively without any significant change in the control group (*p* > 0.05). The intermediate values were detected in rats treated with ZnNPs after ABM exposure. The changes in the hematological parameters in different experimental groups are shown in Table 1. We observed significant reduction in RBCs, PLT, Hb, and HCT in the animals exposed to AMB alone or co-administrated with ZnNPs compared to the control and ZnNPs groups (*p* < 0.05). Contrastingly, the rats administrated with AMB alone or in combined with ZnNPs exhibited significantly elevated WBCs and lymphocytes compared to other groups (*p* < 0.05). There were no significant changes in the MCH, MCHC, and MCV percentages among the experimental and control groups. Generally, the ZnNPs group exhibited improved hematological parameters along with the control group.

**TABLE 1:** Hematological parameters and blood indices values of control and experimental rat.

Parameters	Group I	Group II	Group III	Group IV	*p*-value
RBCs (10^12^/L)	2.60 a	1.85 c	1.88 c	2.3 b	***
Platelet (PLT) (10^9^/L)	77.67 a	47.67 b	46.67 b	67.33 a	***
Hemoglobin(Hb) (g/L)	8.71 a	6.87 b	7.14 b	8.64 a	***
Hematocrit (HCT) %	36.12 a	31.31 b	31.42 b	35.44 a	***
WBCs (10^9^/L)	6.97 c	11.57 a	8.80 b	6.75 c	***
Lymphocytes (%)	78.24 b	82.82 a	81.97 a	79.54 b	**
MCHC (g/dl)	24.11	25.91	22.69	17.63	ns
MCV (fL)	138.99	131.85	133.93	139.31	ns
MCH (pg)	33.46	40.22	36.51	35.05	ns

Different letters represent significant differences (Duncan’s test significant difference test at *p < 0.05, **p < 0.01, and ***p < 0.001 among all treatments.

RBC, red blood cell; WBC, white blood cell; MCHC, mean corpuscular hemoglobin concentrations; MCV, mean corpuscular volume; MCH, mean corpuscular hemoglobin.

Group I, Control; Group II, Abamectin (ABM); Group III, ABM + ZnNPs; Group IV, ZnNPs.

### Effects on antioxidant capacity and oxidative biomarkers

**FIGURE 3:**
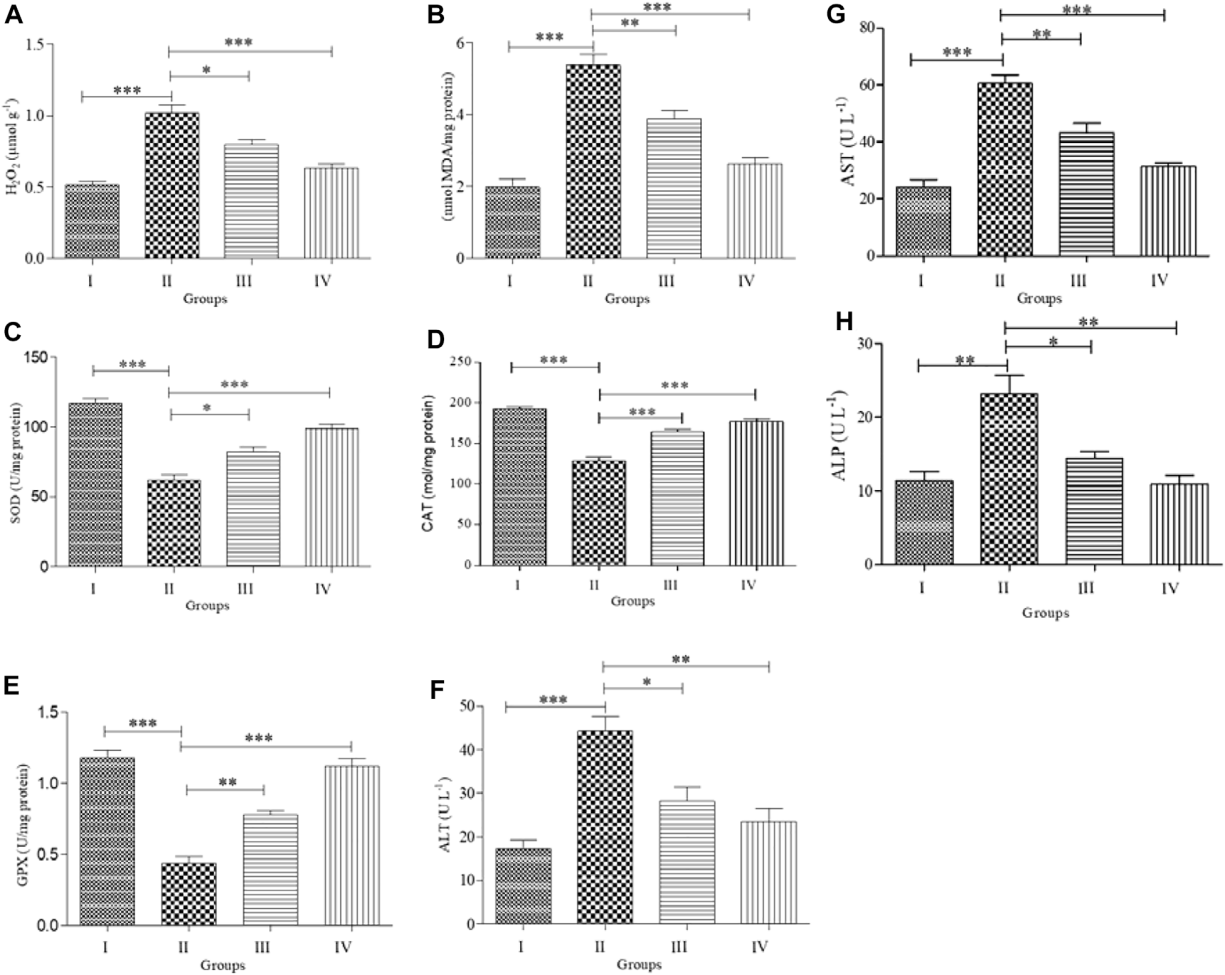
Effects of Abamectin (ABM; G II) and ZnNPs (G IV) alone and together (G III) on oxidative stress and antioxidant markers on serum blood of rats (*n* = 5) compared with control (G I). H_2_O_2_ = hydrogen peroxide **(A)**; MDA = malondialdehyde **(B)**; SOD = superoxide dismutase **(C)**; CAT = catalase **(D)**; GPx = glutathione peroxides **(E)**; ALT **(F)**; AST **(G)**; ALP **(H)**. Each measurement was done at least in triplicate samples and data represented as mean ± SD. Statistical analysis was done using one-way ANOVA for treatments between four groups followed by Tukey’s post hoc test. *p < 0.05 vs. G II, *p < 0.05, **p < 0.01, and ***p < 0.001 vs. G II. Group I: Control, Group II: Abamectin (ABM), Group III: ABM + ZnO, Group IV: ZnO.

We observed statistically significant differences in the levels of antioxidant enzymes (SOD, CAT, and GPx) and oxidative stress biomarkers (MDA and H_2_O_2_) in the serum of treated animals compared to control animals (Figures 3A–E). There was no significant difference in the MDA and H_2_O_2_ levels between the control and ZnNPs-treated rats (Figures 3A–C). However, both SOD and CAT activities (Figures 3D,E) were slightly lower than those in the control group. The ABM-treated rats displayed significantly lower SOD, CAT, and GPx activities while the MDA and H_2_O_2_ levels were significantly elevated. In the ZnNPs + ABM-treated rats, the SOD, CAT, and GPx levels were lower while MDA and H_2_O_2_ were higher compared to normal values. However, these levels were reasonably higher compared with those in Group I and Group II.

### Effects on hepatic function biomarkers

ABM-treated rats displayed significantly higher levels of ALP, AST, and ALT activities compared with Group I (*p* < 0.05). Furthermore, ABM + ZnNPs-treated rats showed significantly reduced ALT, ALP, and AST activities compared with the control and ABM-treated groups (*p* < 0.05) (Figures 3F–H).

### Histopathological findings

**FIGURE 4:**
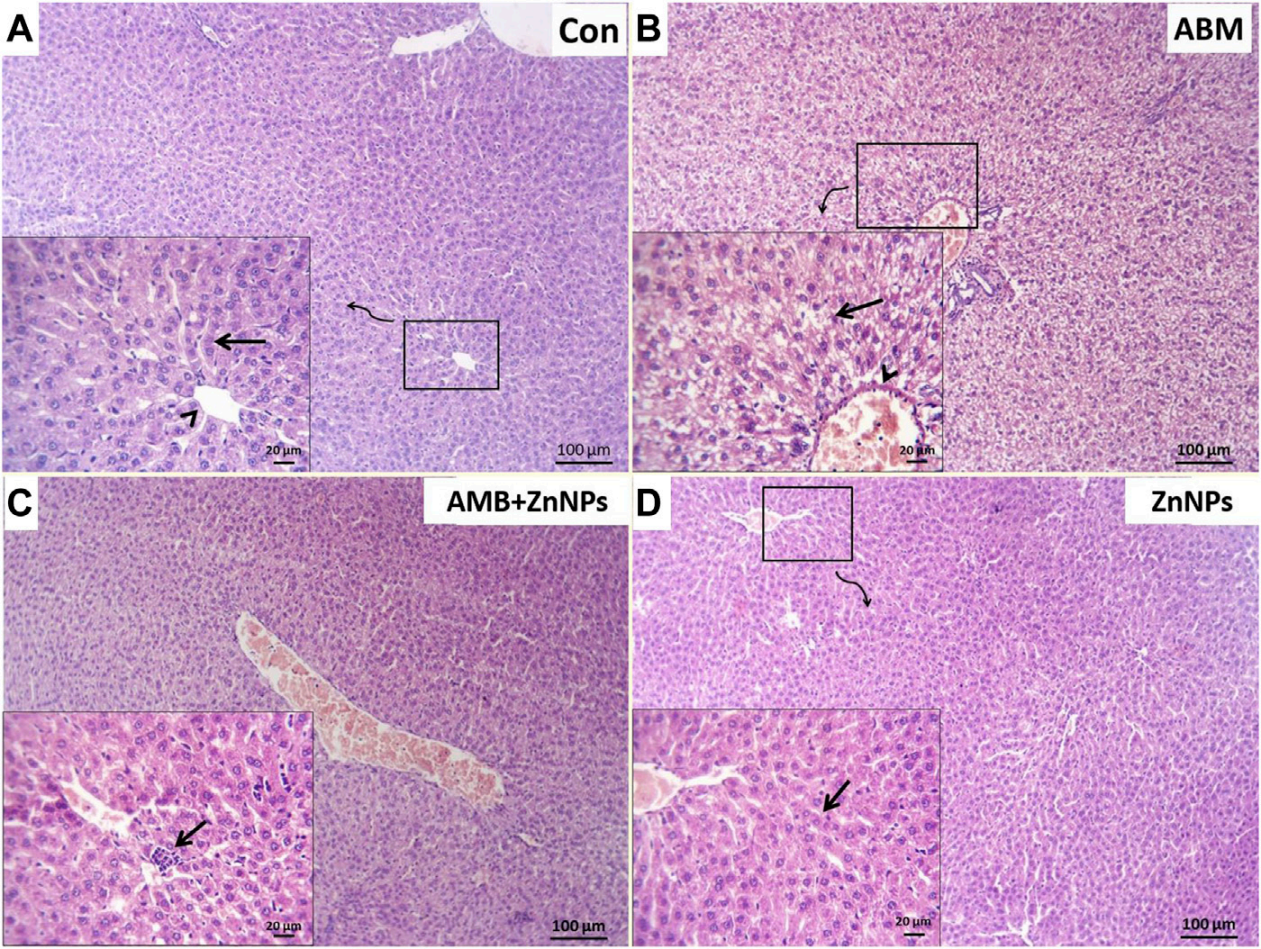
Photomicrograph of H&E stained sections from liver showing some parameters (degenerative changes, necrotic changes, congested blood vessels and round cells infiltration). **(A)**: Normal central vein (arrowhead) and hepatic cords (arrow) in control (G I). **(B)**: Severe degenerated and necrotic hepatocytes (arrow) with congested hepatic blood vessel (arrowhead) in rats group treated with abamectin (ABM; G II). **(C)**: Congested blood vessels and focally infiltration with minute round cells (arrow) in rats group treated with ABM + ZnNPs (G III). **(D)**: Apparently normal histological structures of hepatic tissue (arrow) in rats group treated with ZnNPs (G IV). Scale bar 100 μm for large figures and 20 μm for the small included figures.

**FIGURE 5:**
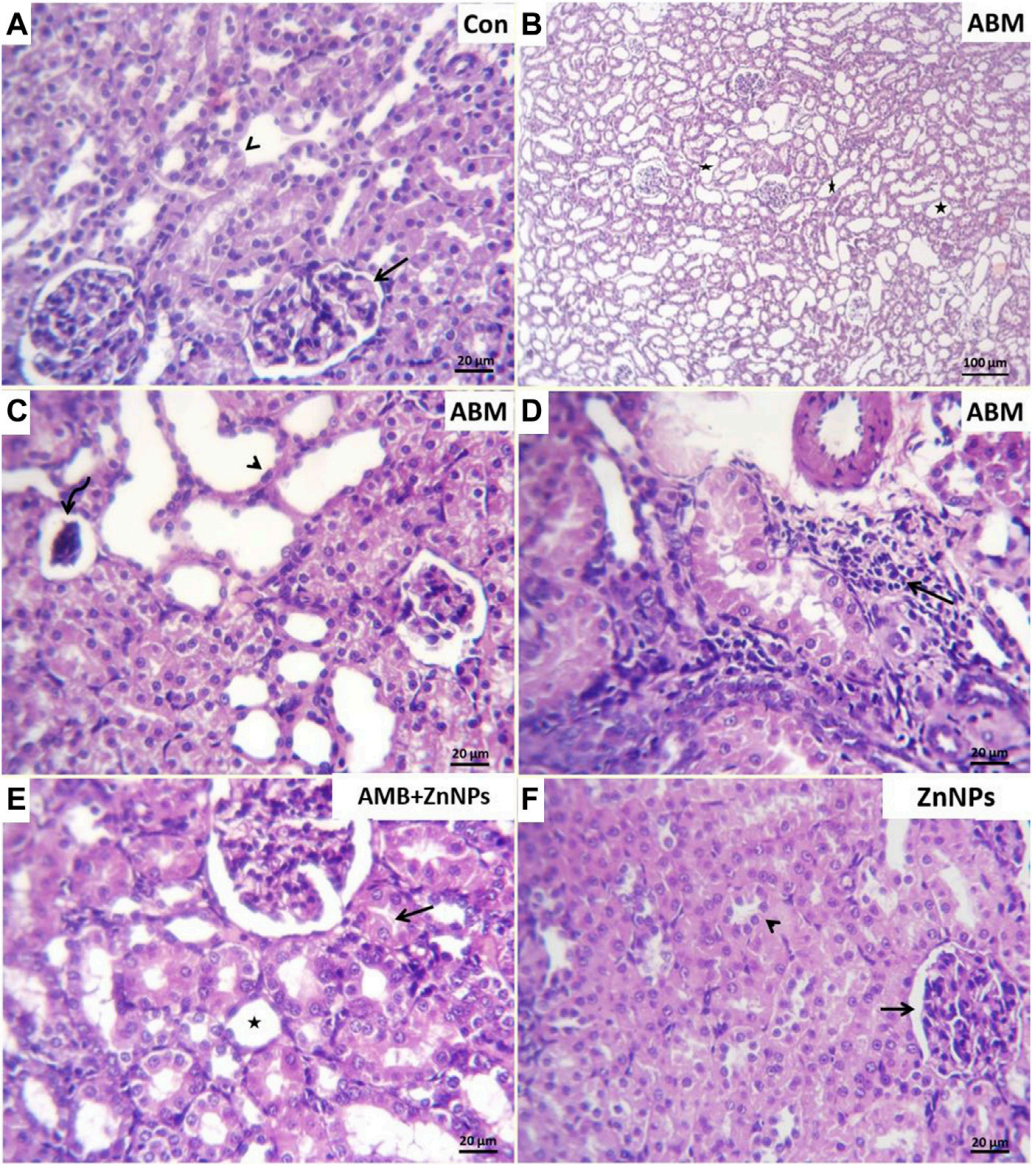
Photomicrograph of H&E stained sections from kidney showing some parameters (Dilated tubular lumina, glomerular shrinkage, degenerative changes, necrotic changes, round cells infiltrations). **(A)**: Normal glomerular architectures (arrow) and surrounding renal tubule (arrowhead) in control (G I). **(B)**: Marked dilated lumina (stars), **(C)**. flattened epithelial lining (arrow head) in most renal tubules, glomerular tufts shrinkage (curved arrow), **(D)**: perivascular round cells infiltration (arrow) in rats group treated with abamectin (ABM; G II). **(E)**: Few tubular dilatation (star) and degenerative changes within some renal tubular epithelium (arrow) in rats group treated with ABM + ZnNPs (G III). **(F)**: Apparently normal glomerular corpuscle (arrow) and renal tubule (arrowhead) in rats group treated with ZnNPs (G IV). Scale bar 20 μm for all figures except 100 μm for panel **(B)**

Histopathological analysis of the liver and kidney tissues was conducted for all the experimental groups. The livers of both control and ZnNPs-treated rats were histologically normal without any aberrant alterations (Figures 4A,D). However, those of the ABM-treated rats exhibited severe degenerated and necrotic changes in the hepatic cords accompanied with congested blood vessels (Figure 4B). The livers of the ABM + ZnNPs-treated rats demonstrated clear and apparent reversal of the histological abnormalities detected in the ABM-treated rats (Figure 4C). The kidney tissues of the control and ZnNPs-treated rats showed normal features with no histological changes (Figures 5A,F). In ABM-treated rats, most of renal tubules showed marked dilated lumina associated with flattened epithelial lining (Figure 5B). Moreover, shrinkage of some glomerular tufts, (Figure 5C), degenerative and necrotic renal tubular epithelium and perivascular round cells infiltration were also detected (Figure 5D). In ABM + ZnNPs-treated rats, the kidneys showed few tubular dilatations and degenerative changes within some tubular epithelium (Figure 5E).

### Immunohistochemical findings

**FIGURE 6:**
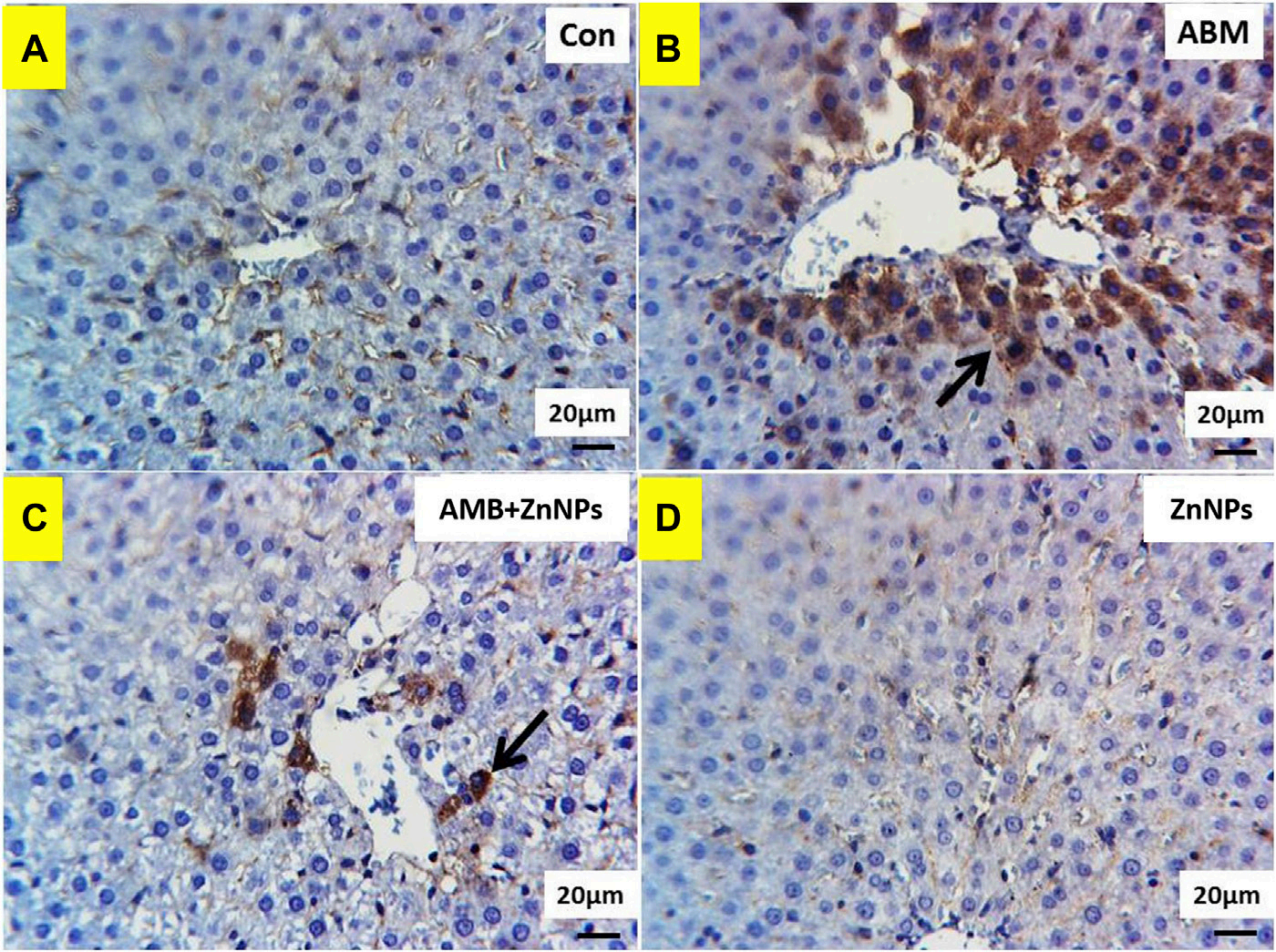
Photomicrographs of Cox2 immunohistochemistry staining in hepatic sections showing: **(A,D)** negative expressions of Cox2 in control (G I) and ZnNPs (G IV), respectively. **(B)** Massive immunoexpression of Cox2 (arrow) in abamectin (ABM; G II) treated rats group. **(C)** Reduced immunostaning of Cox2 (arrow) in ABM + ZnNPs (G III) treated rats group. IHC counterstaining with Mayer’s haematoxylin. Scale bar 20 μm.

**FIGURE 7:**
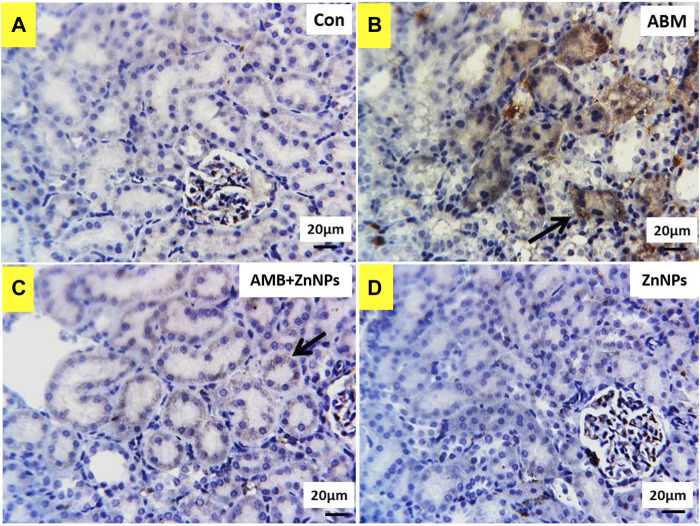
Photomicrographs of Cox2 immunohistochemistry staining in kidney showing: **(A,D)** negative expressions of Cox2 in control (G I) and ZnNPs (G IV), respectively. **(B)** Massive cytoplasmic immunoreactivity of Cox2 (arrow) in abamectin (ABM; G II) treated rats’ group. **(C)** mild to moderate immunostaining of Cox2 (arrow) in ABM + ZnNPs (G III) treated rats’ group. IHC counterstaining with Mayer’s haematoxylin. Scale bar 20 μm.

IHC was used to detect the localization of COX-2 antigens in the liver and kidney tissues of rats in all four experimental groups. The tissues of the control and ZnNPs-treated rats displayed negative staining for COX-2 (Figures 6A,D, 7A,D). On the other hand, the IHC findings for COX-2 in the ABM-treated rats and the ABM + ZnNPs- treated rats revealed the following. As shown in Figure 6B, liver of ABM-treated rats illustrated a massive immunoexpression for the existence of COX-2 antigen which was detected diffuse positive cytoplasmic expressions (golden brown in color) of COX-2. On the other hand, reduced immunostaining of COX-2 in ABM + ZnNPs treated rats’ group (Figure 6C). In kidney, massive cytoplasmic immunoreactivity for the detection of COX-2 antigen in ABM-treated rats is illustrated as a diffuse positive cytoplasmic expression (golden brown in color) of COX-2 (Figure 7B). While the kidney of rats treated with ABM + ZnO treated rats showed mild to moderate immunostaining of COX-2 antigen (Figure 7C).

## Discussion

In veterinary sciences, pesticides are widely used globally as anthelmintic for treating animal diseases, which consequently compromises the health of animals and humans, and also contaminates the environment (Kalyabina et al., 2021). ABM has been shown to adversely affect the antioxidant system and generate oxidative stress in animals, leading to several diseases. Previous studies have shown that several bacteria can be used to synthesize ZnNPs (Yusof et al., 2020) and the microorganism type determines the physicochemical properties of ZnNPs (El-Saadony et al., 2021b; El-Hack et al., 2021; Reda et al., 2021). Here we synthesized ZnNPs using *Lactobacillus* fermentum TA20 supernatant. The synthesized ZnNPs were evaluated using several techniques and were found to be spherical and monodisperse. UV–Vis spectrum has been shown to be essential for identifying and characterizing nanoparticles. Reda et al. (2021) found that ZnNPs manufactured using *Bacillus subtilis* AM12 showed an absorption peak at 320 nm. Moreover, using TEM analysis, Reda et al. (2021) found that the size of the ZnNPs obtained using *Bacillus subtilis* AM12 were 22–43 nm and FTIR spectrum showed the presence of active groups such as alcohols, phenols, and alkenes, which are responsible for stabilizing the nanoparticles. DLS results confirmed that the size of ZnNPs was 25.31 nm with negative charge of −28.7 mV.

Several environmental contaminants have numerous negative health consequences on humans and animals, particularly in developing countries (Manisalidis et al., 2020). When administered at a sub-lethal dose (1 mg/kg), ABM proved non-toxic in the test animals. However, we showed that ABM-treated rats showed significant reduction in BWG compared to the untreated group as shown in Figure 2, indicating potential hazardous effects of this chemical (Bailey et al., 2004). ABM also showed negatively affected the rats’ appetites and BWG by triggering oxidative stress, as shown earlier by Meligi and Hassan (2017). Similarly, in mice exposed to sub-lethal ABM dose, significant reduction in body weight was seen compared to the untreated group at the same level (*p* < 0.05) (El-Gendy et al., 2015), which was restored after co-administration of ZnNPs at 1 mg/kg BW. This data is consistent with that reported by Hassan et al. (2017) who showed that administration of ZnNPs (30 mg/kg BW) into ABM-treated rats nearly normalized their BW, which might be attributed to the regulatory role of ZnNPs in enhancing the antioxidant activity and further increasing the secretion of digestive enzymes.

Blood profile can be used as a health indicator in mammals and fishes, and is also used as a xenobiotic toxicity index (Sancho et al., 2000). Here, we found that ABM-treated rats showed significantly lower RBC counts, Hb concentration, HC, and PLT levels (Table 1).

The reduction in both RBC and Hb might be due to the lysis of RBC resulting from ROS-mediated oxidative injury to the cell membranes (Packman, 2001). Consistent with our findings, several previous studies have shown that the avermectins lowered erythrocyte counts and increased leukocyte counts in test animals (Ali, 1990; Chen et al., 2022). Further, our results are also consistent with those seen by Eissa and Zidan (2010) who clarified that ABM at (1/10 LD_50_) considerably lowered hemoglobin and RBC levels in treated groups.

Moreover, higher WBC and lymphocytes counts seen after ABM treatment clearly indicate active immune response in the animal, possibly due to pesticide-induced tissue damage and necrosis (Meligi and Hassan, 2017). Moreover, increased WBC and lymphocytes might indicate bone marrow depletion. Additionally, the lack of significant differences in the MCH, MCHC, and MCV counts seen in Group II, compared to the untreated group, indicates that ABM does not cause macrocytic anemia as shown by absence of enlarged erythrocytes.

The overproduction of free radicals resulting from exposure to harmful compounds might reduce antioxidant enzyme levels and degrade the redox state, resulting in a decline in cellular defense (Seif et al., 2021). SOD, CAT, and GPx are considered as the antioxidant enzymes involved in cellular defense and are critical for protecting cells against any ROS-mediated environmental stressors. SOD can convert the superoxide anion radical to H_2_O_2_, which is then cleaved into H_2_O and O_2_ (Scassellati et al., 2020). GPx is essential for defending against oxidative damage in important intracellular molecules as it reduces hydroperoxides to water (Rao and Shaha, 2000). MDA is a commonly used biomarker for determining oxidative damage in the cell. It is produced after lipid peroxidation, and often exacerbates oxidative damage (Pizzimenti et al., 2013). Our findings show significant reduction in CAT, SOD, and GPx activities and enhanced MDA and H_2_O_2_ levels in ABM-treated rats, indicating that ABM causes oxidative stress by creating hydroxyl radicals, superoxide anions, nitric oxide, and hydrogen peroxide, all of which contribute to lipid peroxidation (Mansour et al., 2017). Moreover, our results were validated by the observation by Nars et al. (2016) and Meligi and Hassan (2017); ABM reduces antioxidant indicators such as GPx, CAT, and SOD, while increases MDA levels. Contrastingly, SOD, CAT, and GPx activities were remarkably increased in ZnNPs only and ZnNPs + ABM-treated rats. Our findings are consistent with that of a recent study (El-Saadony et al., 2021a) showing that rats administered with ZnNPs and cyclophosphamide display higher levels of CAT activity than when they were given cyclophosphamide alone.

According to our results, ABM significantly increased the activity of ALT, AST, and ALP in the liver. As these enzymes are specific indicators of liver disease (Harper, 1979), their raised levels in the bloodstream indicates hepatocellular damage. Our findings are consistent with those of Djordjevic et al. (2011) who found that the liver, as a major organ, is responsible for xenobiotic detoxification, metabolism, and energy generation. Hence, our results showing enhanced ALT and AST levels in ABM-treated rats reveal that ABM toxicity causes liver damage (Nars et al., 2016). Moreover, Eissa and Zidan (2010) who described that ABM-mediated lowering of ALP and AST levels might be attributed to loss in hepatic function. Furthermore, Meligi and Hassan (2017) showed that ABM negatively impacts liver function and alters the histopathological architecture of liver cells. Hence, these results are consistent with our findings, showing that significantly higher AST levels are seen in ABM-treated rats compared to control rats. Co-treatment of ZnNPs with ABM or ZnNPs alone nearly restored the ALP, AST, and ALP levels to that of the control. These findings are consistent with that obtained by Mansour and Mossa (2010) showing that co-administration of Zn with chlorpyrifos decreased ALT, AST, and ALP activity compared with rats treated with chlorpyrifos only.

Interestingly, our histopathological findings showed lack of harmful effects in both the control and ZnNPs-treated rats, indicating that ZnNPs might boost antioxidant activity and reduce xenobiotic-induced free radical levels (Atef et al., 2016). In ABM-treated rats, we clearly and repeatedly observed severe deterioration and necrotic changes in hepato-renal tissues along with congestion of blood vessels. These results are consistent with those of (Abd-Elhady and Abou-Elghar, 2013) who found substantial diffuse necrosis of renal-hepatic tissues in ABM-treated rats. Moreover, Jenčič et al. (2006) showed that histological changes seen in the kidneys and, to a lesser degree, in the liver, indicate direct toxicity of ABM. Additionally, ABM caused dilated veins, hemorrhagic spots, and degenerative hepatocytes in the liver tissues (Khaldoun-Oularbi et al., 2013). The negative histopathological changes were reversed in the examined hepato-renal tissues of the ABM + ZnNPs treated rats, indicating that ZnNPs had a cytoprotective effect and safeguarded the cells from the damaging effects of xenobiotics by scavenging free radicals (Atef et al., 2016). Our findings were consistent with the report by Essa et al. (2019) showing that ZnO NPs immediately ameliorated the adverse effects after chlorpyrifos uptake and blocked its harmful effects on the immune system. The adsorption process is caused by the electrostatic attraction between negatively charged pesticide anions and the positively charged sorbent surface. As Zn has antioxidant properties, it can protect the cells from oxidative damage caused by xenobiotics (Powell and Hahn, 2000).

COX-2 is an important enzyme involved in hepato-nephrotoxicity pathophysiology. It is widely accepted to induce inflammatory processes associated with several liver pathologies. Xanthine oxidase expedites the transition of hypoxanthine to xanthine and xanthine to uric acid. The by-products of this reaction are H_2_O_2_ and ROS, which have a major contribution in the etiology of tissue damage (Osukoya et al., 2021). Our biochemical and histopathology evaluations were validated by the IHC results for COX-2, which show negative staining for the COX-2 in the tissues of the control and ZnO-treated rats. In ABM-treated rats, COX-2 was found in varying degrees of intensity in the liver and kidney tissues while in ABM + ZnO-treated rats, negative immunoreaction for the COX-2 antigen in liver tissues and mild to moderate immunostaining of COX-2 antigen in kidney tissues were observed. Our findings support the hypothesis that COX-2 plays a role in both inflammation and tumor development (Surh et al., 2001; Yu et al., 2006). Moreover, COX-2 expression might be induced by xenobiotics through activation of nuclear factor kappa B (NF-κB) (Senthil and Wang, 2009). Our result is consistent with previous studies showing that NF-κB plays a role in up-regulating COX-2 (Surh et al., 2001). Furthermore, COX-2 protein expression was elevated after DDE and DDD exposure (Dominguez-Lopez et al., 2012). Overall, our hematological, biochemical, histological, and IHC data show that ZnO protect the liver and kidney against ABM-induced renal-hepatic damage through its antioxidant and anti-inflammatory properties. Therefore, there is growing interest in understanding the potential of nanoparticles to modify the effects of environmental toxicants. However, further research is required to illustrate the molecular mechanism behind this potential activity.

## Conclusions

The present study showed that pretreatment with ZnNPs reduced the hemotoxicity, immunotoxicity, and reno-hepatotoxicity caused by administration of sub-lethal dose of ABM in rats. This protective effect is due to the dose-dependent antioxidant and hepatoprotective potential of ZnNPs. As a result, our research suggests that ZnNPs co-administration might ameliorate the harmful effects of long-term exposure to ABM in animals. However, we need to further explore the mechanisms behind the protective role of ZnNPs in ameliorating the harmful effects of ABM on hepato-renal tissues.

## Data Availability

The original contributions presented in the study are included in the article/supplementary material, further inquiries can be directed to the corresponding author.
